# Neuroprotection in glaucoma

**DOI:** 10.4103/0301-4738.73700

**Published:** 2011-01

**Authors:** Sushil K Vasudevan, Viney Gupta, Jonathan G Crowston

**Affiliations:** 1 Centre for Eye Research Australia, University of Melbourne and Glaucoma Unit, Royal Victorian Eye and Ear Hospital, East Melbourne VIC 3002, Australia; 2 Faculty of Medicine, Universiti Teknologi MARA, 40450 Shah Alam, Malaysia; 3 Rajendra Prasad Centre for Ophthalmic Sciences, All India Institute of Medical Sciences, New Delhi - 110 029, India

**Keywords:** Glaucoma, neuroprotection, pathophysiology, pharmacological approach

## Abstract

Glaucoma is a neurodegenerative disease characterized by loss of retinal ganglion cells and their axons. Recent evidence suggests that intraocular pressure (IOP) is only one of the many risk factors for this disease. Current treatment options for this disease have been limited to the reduction of IOP; however, it is clear now that the disease progression continues in many patients despite effective lowering of IOP. In the search for newer modalities in treating this disease, much data have emerged from experimental research the world over, suggesting various pathological processes involved in this disease and newer possible strategies to treat it. This review article looks into the current understanding of the pathophysiology of glaucoma, the importance of neuroprotection, the various possible pharmacological approaches for neuroprotection and evidence of current available medications.

Glaucoma is an optic neuropathy, specifically a neurodegenerative disease characterized by loss of retinal ganglion cells (RGCs) and their axons. In the past, glaucoma was viewed as a disease of raised intraocular pressure (IOP); however, it has become increasingly clear that elevated IOP is only one of the risk factors for this disease.

Recent evidence indicates that lowering IOP does not prevent progression in all patients and that progression can continue despite effective lowering of IOP. This was clearly depicted in the Advanced Glaucoma Intervention Study (AGIS) trial,[1,2] Collaborative Normal Tension Glaucoma Study,[3,4] the Collaborative Initial Glaucoma Treatment Study Trial,[5] and the Early Manifest Glaucoma Trial.[6] As RGCs cannot divide and regenerate, optic nerve damage is irreversible. It is therefore imperative that these cells are kept alive.

The term neuroprotection refers to mechanisms within the nervous system, which protect neurons from apoptosis or degeneration, for example, following a brain injury or as a result of chronic neurodegenerative diseases. It has been a common approach that has been used to treat a variety of chronic neurodegenerative diseases such as Parkinson’s and Alzheimer’s disease, to name a few. Numerous theories of neuroprotection in glaucoma have been drawn from these neurodegenerative conditions, where the loss of the cells is targeted instead of the disease process by which these losses occur. This approach attempts to accelerate or impede specific biochemical pathways that may prevent neuronal injury or accelerate neuronal recovery. Hence, any therapy that prevents, retards or reverses apoptosis-associated neuronal cell death resulting from primary neuronal lesions is neuroprotective.

Therefore, neuroprotection in glaucoma is aimed at protecting those neurons that are damaged or likely to be damaged in glaucomatous optic neuropathy which consists of neurons along the entire visual pathway, chiefly the RGC axons. This strategy is an addition to that achieved by IOP lowering alone. Even though any treatment approach that preserves RGCs in glaucoma could be described as neuroprotective, the term has been limited by many researchers to describe a drug that directly interacts with neuronal or glial elements within the optic nerve head.

Consequently, the endpoint of neuroprotection in glaucoma offers a means to prevent the irreversible loss of those cells in glaucoma, especially where the particular etiology is either idiopathic or differs from patients to patients. Hypothetically, neuroprotection should be beneficial irrespective of the pathophysiology of the disease.[7–9]

## Pathophysiology of Glaucomatous Damage and Importance of Neuroprotection

The pathogenesis of RGC loss in glaucoma remains incompletely understood. A diverse range of mechanisms have been postulated, and whether the site of primary damage is the ganglion cell body or their axons remains disputable. Axonal or white matter diseases include anterior ischemic optic neuropathy (AION) and glaucoma, while central retinal arterial occlusion (CRAO) and high levels of acute IOP are diseases of the cell body or gray matter.[10] In a cell body disease, the cell metabolism is directly affected and for that reason, the window to change the outcome is approximately 45–90 minutes for cell rescue, whereas in axonal injuries, metabolism is indirectly affected, giving a longer window of opportunity, and therapies can hence be delivered after the acute insult.

In glaucoma, there is transsynaptic degeneration as evidenced from experimental primate and human glaucoma, which suggests that axonal injuries extend from the optic nerve to visual pathways in the brain. Thus, central neurodegenerative changes in the visual pathway may contribute to the pathophysiology of glaucomatous progression.[11] Accordingly, therapies combining IOP-lowering approaches with neuroprotective agents would confer protection of local and central visual neurons, thus preserving vision. In view of the fact that glaucoma is a long-standing disease with features of axonal damage, it is therefore a good candidate for neuroprotection.

### Glutamate induced excitotoxicity

Excitotoxicity has been implicated in almost all chronic neurodegenerative diseases. The amino acid glutamate is an essential neurotransmitter in the central nervous system as well as the retina. Glutamate concentrations higher than the physiological levels are toxic to the neurons depending on the duration and magnitude of the increase. Excitototoxic neuronal injury involves a self-perpetuating cascade of events originating from a persistent activation of ionotropic glutamate receptors.[12] It has been observed that neuronal hypersensitivity to glutamate can occur in certain conditions and, for that reason, elevated glutamate levels are not required to induce neuronal cell injury.

**Figure 1:**
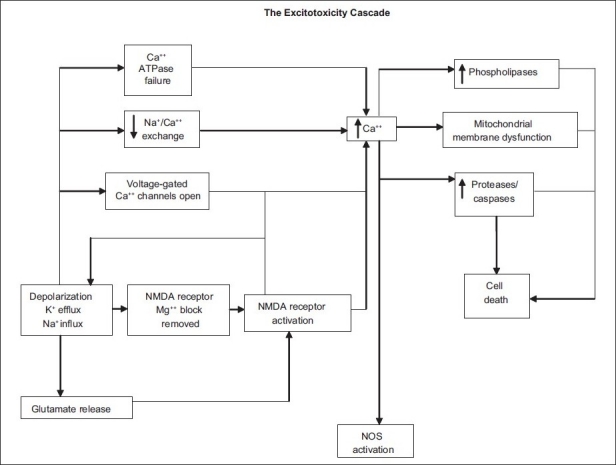
Excitotoxicity cascade leading to neuronal injury or death is caused by excessive exposure to the neurotransmitter glutamate and uncontrolled stimulation of its membrane receptors, mainly the NMDA-sensitive channel (Source: Adriana Di Polo, PhD and Leonard A Levin, MD, PhD. Strategies for prevention of neural injury in glaucoma. In: Levin LA WR, Weinreb RN, Di Polo A, editors. A Pocket Guide of europrotection in Glaucoma. New York: Ethis Communications; 2007. pp.36)

*N*-methyl-D-aspartate (NMDA) receptor activation leads to opening of associated ion channels in the neurons and the entry of extracellular calcium and sodium. Glutamate-mediated neuronal toxicity is dependent on the influx of extracellular calcium, which in turn acts as second messenger to activate a cascade [Fig. 1], consequently leading to neuronal cell death.[13] In order to maintain the physiological concentrations and to protect ganglion cells from excitotoxic cell death, appropriate removal of synaptic glutamate is necessary. Müller cells and astrocytes surrounding the synapses express the glutamate transporter which transports extracellular glutamate into the glial cells. Within the glial cells, glutamine synthetase converts glutamate to glutamine which is non-toxic and is released by glial cells to be taken up by neuronal cells where it is again converted to glutamate in the presence of glutaminase. This cycle replenishes the neurotransmitter stores and glutamate toxicity is averted.

However, it still remains to be ascertained whether glutamate excitotoxicity is an initial response to elevated pressure and ischemia or secondary to glutamate release from dying ganglion cells. While there is substantive evidence that high doses or prolonged exposure to glutamate promotes RGC death via overactivation of ionotropic glutamate receptors,[14] no consensus has been reached in answering the question on whether the level of glutamate is truly elevated in glaucoma.

### Neurotrophin withdrawal

As the nervous system develops, the surplus of neurons produced is subsequently eradicated by apoptosis. Neurons require neurotrophic growth factors, which are acquired by retrograde axoplasmic transport. These growth factors, known as neurotrophins, regulate cellular metabolism hence maintaining the normal cellular milieu.[15] Thus, where neurotrophic support is absent due to retrograde axonal transport block, RGCs die. This group of small growth peptides comprises brain-derived neurotrophic factors (BDNF), nerve growth factors (NGF), neurotrophin-3 (NT-3) and neurotrophin-4 (NT-4).[16,17] Lack of BDNF and NGF secreted by RGC targets results in apoptosis of developing RGC though it is postulated that it has almost no effect on the survival of mature RGCs since retrograde transport of neurotrophin factors persists along adult RGC axons. Hence, in glaucoma, blockade of axonal transport results in neurotrophin deprivation leading to neuronal cell death.[17–29]

### Apoptosis

Apoptosis refers to a common mode of cell death. It is a subtle process where the cell initiates a death program and commits suicide resulting in cell shrinkage, genomic fragmentation and nuclear pyknosis.[30] In necrosis on the other hand, destruction of the cell membrane leads to spillage of cellular contents into the extracellular matrix (ECM), hence damaging and killing other cells. This sequential occurrence of cell death processes appears to be biphasic, and research in optic nerve injury models has shown both fast and slow phases of RGC degeneration.[31] It may therefore be reasonable to theorize that the early phase of degeneration, i.e. from 1 to 3 days, could represent necrosis, followed by apoptosis.

There are many triggering factors for apoptosis, be it extracellular or intracellular events. These include trophic factor deprivation or oxidative damage, both of which have been postulated to induce RGC apoptosis in glaucoma. A principle class of intracellular apoptotic regulators is the Bcl-2 family of mitochondrial membrane-bound proteins. While some proteins in this family inhibit apoptosis (e.g. Bcl-2, Bcl-X_L_), others promote it (e.g. Bax, Bad, Bid).

Caspases are proteases that execute the dismantling and demolition of apoptotic cells. Caspases are categorized into two broad groups: initiators (e.g. caspases 8 and 9) which activate other caspases and effectors (caspase 3) which cleave specific substrates involved in cellular disassembly. Some experimental glaucoma models have shown that the initiator caspases are activated, while inhibition of the effector caspases can be neuroprotective.[32–40] Calcium overload is also responsible for activation of calpane and caspase cascades, leading to apoptosis.[41]

### Nitric oxide

Nitric oxide (NO), a free radical which is formed from l-arginine by NO synthetase (NOS), is thought to play a role in many neurodegenerative diseases including glaucoma, Alzheimer disease, multiple sclerosis and cerebral-cardiovascular diseases.[42] There are three isoforms of NOS: NOS-1 neuronal, a constitutive enzyme that been detected in the diminished nerve fiber bundles at the prelaminar region and lamina cribrosa of glaucomatous eyes;[43] NOS-2, an inducible enzyme (iNOS) produced in response to high IOP, which also has a genetic association in patients with primary open angle glaucoma (POAG); and NOS-3 endothelial, another constitutive enzyme found in the prelaminar region of the optic nerve, which functions as a vasodilator.[44]

It has been proposed that damage to optic nerve axons at the level of lamina cribrosa is caused by excessive NO production by reactive astrocytes and microglia in the optic nerve heads. Studies have shown that NOS-1 and NOS-3 are upregulated in POAG patients, and NOS-2, absent in healthy individuals, is expressed in the optic nerve head of POAG patients. Excitation of NMDA receptors has also been shown to trigger NOS-1, which in turn upregulates NOS-2, leading to a large increase in cellular NO level, while induction of NOS-3 can provide neuroprotective effects by causing vasodilatation and increased blood flow to the optic nerve head.[43–46]

A number of pathogenic insults including ischemia, reperfusion, inflammation and excitotoxicity have been linked with raised levels of NO in the retina. NO has the ability to pass from one neuron to another bypassing synapses, leading to rapid intra and intercellular diffusion. Being a free radical with moderate activity, it causes the formation of free radicals that contribute to RGC demise.[47–50]

### Free radical generation and oxidative stress

Free radicals are a byproduct of oxidative metabolism. The high metabolic activity of retinal tissues render RGCs especially vulnerable to oxidative stress.[51] Free radicals interfere with macromolecular cellular constituents of the cells and further lead to derangement of protein breakdown, lipid peroxidation and nucleic acid degeneration, resulting in cell death.[52] To counteract this, ocular tissues have highly efficient antioxidant mechanisms that include the superoxide dismutase-catalase system, ascorbic acid and reduced glutathione.

### Calcium-dependent pathways

Calcium is an important cation that is usually seized by the mitochondria, endoplasmic reticulum and a number of calcium binding proteins. High levels of calcium are toxic to cells. Three different calcium channels exist: voltage-sensitive calcium channels, store-operated channels and receptor-operated channel such as NMDA receptor. Neuronal death and axonal degeneration are associated with an increase in intracellular and intra-axonal calcium. Calcium-induced neuronal apoptosis is dependent on calcineurin which is a calcium-dependent phosphatase and facilitates the dephosphorylation of the pro-apoptotic mitochondrial membrane-bound proteins i.e. Bad. The dephosphorylation in turn results in its translocation from the cytoplasm to the mitochondria, where it binds to Bcl-2 or Bcl-X_L,_ forming protein complexes, which raise mitochondrial membrane permeability, release cytochrome C and cause neuronal suicide.[53–55]

### Structural abnormalities

The survival of RGC can be jeopardized by changes in the ECM. Matrix metalloproteinases (MMP) degrade ECM proteins. Increased activity of MMP and decreased ECM proteins such as laminin has been observed in the RGC layer in experimental glaucoma models. MMP upregulation may result from high IOP itself, secondary mechanical damage from raised IOP or upregulation of glutamate receptors.[56,57]

### Heat shock proteins

Heat shock proteins (HSPs) or stress proteins are expressed in most cells under normal physiological conditions. They play an important role in normal cellular function such as cellular protein maturation. They are also synthesized in large quantities in response to environmental stresses such as heat, anoxia, and exposure to cytokines and are believed to play an important role in re-establishment of normal cellular function and protect against further damage. They have been found to be highly expressed in the eyes of glaucoma patients and animals with chronic ocular hypertension and are hence thought to be a form of an endogenous defense mechanism against trauma. Though they may serve initially to protect cells from further destruction and facilitate repair, there are evidences suggesting that they may later recruit immune responses that contribute to the progression of disease.[58]

### Immunology

Studies have been carried out to examine ways of manipulating the immune system to preserve RGCs. One approach has been to determine the effect of focal activation of the immune system in the optic nerve. These studies demonstrated that activated T-lymphocytes that have been primed to the constituents of the optic nerve, such as the myelin basic protein (MBP), would be drawn to areas of injury and release neuroprotective factors. To elucidate this, optic nerve partial crush models were used, where many axons were only partially damaged. This approach was later tested in laser-induced ocular hypertension models in rats with parallel effects. Additionally, it has been learnt that this immune-mediated protection can also be generated by glatiramer acetate (copolymer-1), a synthetic polypeptide.[59–62]

### Vascular insufficiency

Impaired blood flow and disruption of autoregulation of the optic nerve blood flow are thought to contribute to the pathogenesis of glaucoma. Vascular insufficiency is also associated with elevated endothelin-1 levels in the aqueous humor and plasma. Endothelin-1 is a potent vasoconstrictor that further compromises blood supply to retinal tissues.[63–65] The resultant ischemic damage triggers astrocytes and microglia to produce tumor necrosis factor (TNF)-α, which leads to apoptosis via caspase-8, indirectly. The interaction of TNF- signaling with other cellular events in glaucomatous neurodegeneration plays a role in the spread of neuronal damage by secondary degeneration. Inhibition of TNF-α death receptors signaling may be a possible treatment modality providing neuroprotection.[66]

### Rationale for neuroprotection

The path to clinical use of neuroprotectants has been long and uneven. Although the possibility of non–IOP-lowering therapy for glaucoma was first recognized in 1972 by Becker *et al*. with the use of diphenylhydantoin for treatment of visual field loss in primary open-angle glaucoma, only of late significant advances have been made in the understanding of the mechanisms for death of retinal neurons.[67]

The randomized clinical glaucoma trials have demonstrated progression of the disease despite significant pressure lowering. A retrospective subanalysis of the AGIS data showed that variation of IOP readings across office visits was more important than the absolute level.[68] Asrani *et al*. suggested that the diurnal IOP range and range over multiple days were significant risk factors for progression, even after taking into account office IOP, age, race, gender, and visual field damage at baseline.[69] Thus, glaucoma will progress even in patients with effective IOP lowering, rendering it a good candidate for neuroprotection.

## Pharmacological Approaches

### NMDA receptor antagonists

As described in the pathophysiology above, excess glutamate leads to NMDA receptor overactivation as well as excitotoxicity. Hence, using NMDA antagonist would be an efficient way to prevent RGC loss where excitotoxicity is implicated.[14] The earliest experiments used MK-801 which completely blocks normal glutamatergic neurotransmission, which is required for normal central nervous system function, and is therefore inappropriate for clinical use.[70] Experimental models of retinal ischemia induced by transient elevation of IOP have shown that NMDA inhibition with MK-801 confers neuroprotection by decreasing the expression of Bad and transient deactivation of the pro-survival kinase Akt pathway.[71]

The development of NMDA blockers that discriminate excessive NMDA receptor activation without affecting normal function led to clinically viable antagonists. Memantine is a non-competitive, low-affinity, open channel blocker. It exhibits selective blockade of the excessively open channels with a fast-off rate, thus inhibiting excessive NMDA receptor activity while maintaining normal neuronal cell function as it does not accumulate significantly within the channel.[72,73] Memantine, being well tolerated, has been approved for its use in Alzheimer’s disease and vascular dementia as it is a highly effective neuroprotective agent as demonstrated in acute animal models of RGC death.[74]

**Figure 2:**
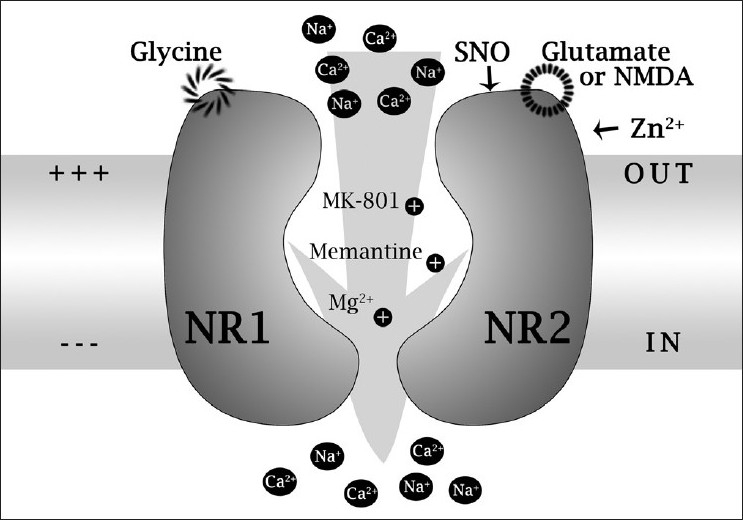
NMDA receptor model illustrating binding and modulatory sites. Glu/NMDA: glutamate/NMDA binding site; Gly: glycine binding site; Zn2+: zinc binding site; NR1 and NR2: NMDA receptor subunits 1 and 2A; SNO: cysteine sulfhydryl reacting with nitric oxide species X (Mg2+, MK-801, and memantine binding sites within the ion channel pore region) (Source: Lipton SA. Paradigm shift in NMDA receptor antagonist drug development: molecular mechanism of uncompetitive inhibition by memantine in the treatment of Alzheimer’s disease and other neurologic disorders. J Alzheimer’s Dis 2004; 6:S61-S74)

The largest randomized, progressive, Phase 3 clinical trial on neuroprotection studying the safety and efficacy of memantine for open angle glaucoma has been completed, but it disappointingly failed to meet its primary endpoint [Fig. 2].[75,76]

### Neurotrophic factors

Various studies in experimental models have shown that neurotrophic factors, especially BDNF and ciliary neurotrophic factor (CNTF), can enhance survival of RGCs after optic nerve injuries but there are to date no adequately powered clinical trials to substantiate this in humans.[77–79] Recent studies have shown that a combination of BDNF and LINGO-1 (a CNS-specific leucine-rich repeat protein) antagonist enhances long-term RGC viability. Most of the investigations have been focused on BDNF, and although results are promising, it still remains inconclusive.[76,80]

### Anti-apoptotic agents

Several pathogenic mechanisms have been proposed to induce apoptotic RGC death in glaucoma. These include reduced neurotrophic factors and cytokine deprivation to neurons, altered intracellular calcium levels, reactive oxygen species and excitotoxicity due to raised extracellular levels of certain neurotransmitters and neuromodulators.[26,81–84] Enhancing mitochondrial function may also inhibit apoptosis. Recent studies have shown that supplements of creatine, α-lipoic acid, nicotinamide and epigallocatechin-gallate (EGCG), which act by counteracting oxidative stress, promote mitochondrial function and confer neuroprotection.[85]

Two approaches for inhibiting apoptosis have been proposed. The first method is to promote survival pathways. For instance, the use of brimonidine activates anti-apoptotic extracellular signal regulated kinase (ERK) and Akt, which in turn enhance the production of Bcl-2 and Bcl-X_L_.[86] A second approach is to block the apoptotic machinery by using caspase inhibitors.[87–91] Caspases are the effector enzymes that disassemble cellular contents during apoptosis. Calpeptin, a calpain-specific inhibitor, has been shown in glaucoma experimental models to confer neuroprotection.[92] As other death processes such as autophagy and necrosis may also play a role in RGC death in glaucoma, inhibition of apoptosis alone may not completely prevent glaucoma progression.[76]

### Nitric oxide synthase antagonists

Inhibition of NOS using 2-aminoguanidine, i-NOS and L-N[^6^-(1-iminoethyl) lysine 5-tetrazole amide has been shown to be neuroprotective in experimental glaucoma models.[93] Nipradilol, a β- and α_1_ antagonist has also been shown to be neuroprotective.[94,95] However, Pang *et al*., in a recent study, found no proof for the release of NOS-2 by astrocytes in patients or models. In addition, they documented that there were no neuroprotective properties in aminoguanidine.[76,96]

### Antioxidants

RGC death by NMDA-induced toxicity may be reduced by antioxidants and free radical scavengers such as vitamins C and E (α-tocopherol),[97,98] superoxide dismutase and catalase.[52,76] Gingko *biloba* (EGb761), apart from increasing blood flow, has been also found to have a free radical scavenger property.[99] Its extract is also known to preserve mitochondrial metabolism and enhance ATP production in various tissues.

### Calcium channel blockers

Calcium channel blockers like nifedipine and verapamil may exert neuroprotection by increasing blood flow to the RGCs.[100] In addition, they also improve glutamate metabolism and hence cause efficient homeostasis in the optic nerve head.[101] However, there are concerns that by also causing systemic hypotension these agents can worsen retinal ischemia due to a reduction in perfusion pressure.

A recent study in a rat chronic glaucoma model has shown that continuous treatment using candesartan (angiotensin II type I receptor blocker) provided substantial neuroprotection against RGC loss.[76,102]

### Gene therapy

The current core of gene therapy is targeted against apoptotic factors. Candidate agents are deprenyl, a monoamine oxidase inhibitor (anti-parkinsonism drug) which increases the gene expression of factors that halt apoptosis, and flunarizine and aurintricarboxylic acid, which have shown promising results in retarding apoptosis following light-induced photoreceptor cell death.[76,103]

### Immunomodulators and vaccination

The objective of vaccination is not only to ameliorate disease propagation but also to decrease the secondary degeneration of neurons following the acute insult. Passive transfer of T cells specific to MBP is one such approach. In order to withstand any insult, the retina and optic nerve require an intact peripheral immune system. These T cells are required to identify site-specific self-antigens. They activate resident microglia and harness blood borne monocytes which have been shown by some investigators to support regrowth of axons and arrest degeneration. Glatiramer acetate (copolymer-1/cop-1), a synthetic oligopeptide, is being studied as a possible vaccine used for neuroprotection.[104–106]

The locally activated anti-self T cells target the injury and supply cytokines and growth factors which govern sentinel cells, microglia and enlist macrophages bequeathing the eye with a protective phenotype. These cells arrest the production of TNF-α, as well as remove glutamate and debris and generate growth factors.[59,107–112]

### Geranylgeranylacetone

As discussed earlier, the role of the HSPs in the pathophysiology of glaucoma has led to evaluation of geranylgeranylacetone (GGA) which is used clinically in peptic ulcer disease. It has been observed to evoke the synthesis of HSP70, thus rendering it potentially neuroprotective.[76,113]

### Stem cell therapies

Stem cell transplantation is another promising modality being researched for many neurodegenerative diseases. Stem cells are thought to exert neuroprotective effects by generating neurotrophic factors, modulating MMP and other aspects of the CNS environment that may promote endogenous healing.[114] Research on stem cell mobilization and the possible neuroprotective contribution of granulocyte-colony stimulating factor (G-CSF) showed that G-CSF was greatly expressed by the RGCs, thereby providing neuroprotection in neurodegenerative diseases.[115] Also, oligodendrocyte precursor cells (OPCs), a type of neural stem cell, may provide protection to RGCs from damage.[76,116]

### Bioenergetics

Bioenergetics is the study concerning metabolic processes that lead to energy utilization in the form of ATP molecules. Emerging evidence points that energy failure and mitochondrial dysfunction at the optic nerve head (ONH) may be involved in glaucoma due to reduced energy and increased free radical production.[117]

Enhancing mitochondrial function or increasing energy supply of neurons may provide an additional method for inducing neuroprotection. Such approaches have been successful in animal models of other neurodegenerative disease including Parkinson’s disease and traumatic brain injury. They act by increasing energy buffering capacity of damaged cells which decreases permeability of mitochondrial membrane pore and free radical scavenging. These approaches remain unexplored in glaucoma models.[76,118]

## Evidences on Currently Available Topical Medications

A number of large prospective randomized controlled trials have demonstrated the impact of IOP lowering on inhibiting glaucoma progression or preventing conversion of ocular hypertension to glaucoma. IOP reduction is achieved by inhibiting aqueous humor flow (influx) or enhancing aqueous outflow. Among the broad categories of drugs available are the α_2_-adrenoceptor agonists, β-adrenoceptor antagonists, prostaglandin derivatives and carbonic anhydrase inhibitors.

### α_2_-Adrenoceptor agonist

α_2_-Adrenoceptors are located in the ganglion cell layer of the retina.[119,120]

Activation of these receptors inhibits neuronal cell death via a complex but independent pathway. There is mounting evidence implicating that α_2_-adrenoceptors inhibit the pro-apoptotic pathway,[86] trophic factor release,[121] as well as glutamate release,[122] providing neuroprotection. They activate phosphatidyl-inositol-3 (PI3) kinase and protein kinase/Akt pathway, which are the major pathways attributed to cell survival. It blocks apoptosis via phosphorylation-dependent inhibition of pro-apoptotic signaling molecules such as Bad and caspase-9, and activation of anti-apoptotic molecules such as NF-kappaB. Stimulation of the α_2_-adrenoceptors also activates ERK and increases the synthesis of survival factors such as bFGF and BCL-2.[123–125]

Recently, it has been shown that α_2_ modulation of NMDA receptor function plays an important role in neuroprotection based on neuromodulation instead of direct inhibition of the NMDA receptor for the treatment of glaucoma and other CNS disorders associated with NMDA receptor overstimulation.[126] Brimonidine, being a highly selective α_2_-adrenoceptor agonist, which lowers IOP essentially by decreasing aqueous humor inflow, has been established to be neuroprotective to RGCs in this manner. There is an ongoing large randomized controlled clinical trial of neuroprotection called the Low-pressure Glaucoma Treatment Study (LoGTS) comparing brimonidine and timolol. However, the results are not yet available.[76,127]

### β-Adrenoceptor antagonists

Another category of widely available drugs is the β-adrenoceptor antagonists, which is further subdivided into the β_1_-selective (e.g. betaxolol) and non-selective (e.g. timolol) β-blockers. All β-blockers lower IOP via inhibition of β_2_-adrenoceptors present on the ciliary epithelium, thus reducing aqueous humor flow. The neuroprotective elements of β-blockers are believed to be mediated by inhibition of calcium and sodium ion influx into neurons, which occurs in hypoxia, ischemia and excitotoxicity. NMDA and glutamate affinity is also reduced, thereby further reducing calcium influx into the RGCs.[128–135] Timolol binds to voltage-gated calcium and sodium channels, which in turn reduces NMDA stimulated calcium influx, however, to a much lower affinity in comparison to betaxolol. Although the systemic route is just as important as the topical route, betaxolol seems to accumulate in membranes as it is highly lipophilic. Hence, concentration is appreciably lower in vitreous or retina. Correspondingly, high doses of timolol are required to be absorbed systemically. Hence, the topical route may have a better efficacy in reducing IOP and RGC loss though absorption of timolol into systemic circulation which plays an equally vital role.[136] To date, there have been no large clinical trials of β-blockers for neuroprotection in glaucoma.[136–139] Though no large randomized clinical trials have been performed, a number of small clinical trials which compare the effects of betaxolol and timolol on visual field progression in patients with POAG have shown that betaxolol seems to confer better visual field preservation though its ability to reduce IOP is less compared to timolol.[76,140–148]

### Prostaglandins

It is well accepted that in the pathogenesis of ischemic and inflammatory injuries, prostaglandins including PGF_2α_ are implicated. They are potent vasoconstrictors and can possibly play a role in the pathogenesis of ischemia and inflammation; however, there is currently no strong evidence to suggest that they are toxic to the retina or optic nerve. Drugs such as latanoprost, travoprost, bimatoprost and unoprostone enhance aqueous outflow, thus reducing IOP. Latanoprost exerts its neuroprotective effects by impeding glutamate and hypoxia-induced apoptosis and is postulated to act via negative feedback on cyclooxygenase-2 activity. Though intravitreal administration of latanoprost has demonstrated an increase in RGC survival following trans-section of the optic nerve, no electrophysiological data have been documented with regard to the mechanism of action. Again, there have been no large clinical trials focusing on the neuroprotective effects of prostaglandins.[76,149–151]

### Carbonic anhydrase inhibitors

Another major group of drugs is the carbonic anhydrase inhibitors, e.g. dorzolamide and brinzolamide, which are selective carbonic anhydrase isoenzyme II inhibitors located in the ciliary epithelium. Carbonic anhydrase isoenzyme II inhibition ensues a reduction in aqueous humor formation as well as increases blood supply to the choroid and optic nerve head, regardless of IOP, though the mechanism is unknown.[152,153] Although it seemed to be neuroprotective in a rat hypertension model, the level of neuroprotection correlated with the level of IOP reduction which might mean that the neuroprotection conferred by these agents may be due to the IOP reduction rather than its direct neuroprotective properties. To date, there is inadequate evidence to show that this group of drugs is successful in providing neuroprotection.[76,154,155]

### Other compounds and alternative therapies

Erythropoietin, being a hematopoietic cytokine, has shown to hold amazing neuroprotective properties in pre-clinical models.[156] Endocannabinoids play a big role in central nervous system neurodegenerative diseases. They have vasodilatation properties giving them neuroprotective effects. There are also many natural compounds like omega-3 fatty acids, carnitine, coenzyme Q10, citicoline, curcumin, danshen (*Salvia miltiorrhiza*) and resveratrol that potentially confer neuroprotection via various mechanisms. However, there are no large clinical trials to date which support the use of these compounds in the treatment of glaucoma.[157] Reflexology, the science of stimulating the body’s healing forces, is known to increase blood circulation[158] and may be helpful to patients with glaucoma.

## Clinical Trials for Glaucoma Neuroprotection

In the past, studies on neuroprotectants have had low success rates in the transition from the laboratory to human trials. Despite successful preclinical cell culture and animal model experiments, most of the Phase 2 and virtually all of the Phase 3 clinical trials of more than 100 neuroprotective drug candidates have failed to demonstrate efficacy, acceptable safety, or patient benefit.

### A) Memantine trial

The two-part memantine trial was designed to test the safety and efficacy of oral memantine as a treatment for glaucoma. The first Phase 3 trial did not meet the primary endpoint, but showed a statistically significant difference in a secondary functional endpoint. The second Phase 3 trial did not replicate the results of the secondary endpoint of the initial trial, and although the study showed that the progression of disease was substantially lower in patients receiving a higher dose of memantine compared to patients receiving the lower dose, there was no statistical significance when compared with patients receiving placebo. Therefore, the study did not meet its primary endpoint and did not sufficiently replicate the results of the initial Phase 3 trial. Additional analyses are still ongoing in compliance with safety and regulatory requirements.[159]

### B) Brimonidine

A small study of topical brimonidine in nonarteritic ischemic optic neuropathy reported that visual fields tend to be slightly better in the treated group than in the untreated group, but the difference was not statistically significant.[160] Treatment with brimonidine is also currently being studied in comparison to timolol in the LoGTS.[127] The common rationale for human trial failures is that the animal models do not properly simulate the human disease or that the variability of the disease in patients is much higher than the variability of the disease in laboratory animals.

The most challenging issue is the endpoint when designing the clinical trial for neuroprotection. There are several endpoints that could be applied and visual function is an important outcome to consider. However, there is no optimum way to measure the visual function due to inconsistent variables. Whether the endpoint to consider should be an event or the rate of progression remains a vital question that needs to be answered in a neuroprotection trial.

## Proposed Guidelines for Use of Neuroprotective Agents

Patients who may benefit from a proven neuroprotectant are suggested to be based upon the stage of the disease.[161] In advanced glaucoma where fixation and/or patient mobility is threatened, and moderate damage with high risk of progression, conventional ocular hypotensive therapy and the neuroprotectant agents should be employed. In glaucoma with moderate damage but low risk of progression, conventional ocular hypotensive therapy should be used and neuroprotective agents should be considered if there is an increase in damage or risk for progression.

## What the Future Has to Offer

Extensive research has been conducted on the pathophysiology of glaucoma with the hope of discovering new treatment modalities to this incapacitating disease. Recently, in 2009, A. Di Polo and Y. Yücel introduced the concept of neuroregeneration. It is defined as any strategy that promotes regrowth of axons or dendrites and restores connections with postsynaptic neurons, thereby restoring cell function. Neurons must first be healthy to regenerate, i.e. neuroprotection must have already occurred despite a stressful milieu. Understanding neuroregeneartion remains an active area of neurobiology research which may help gain insight into neuroprotection and eventually be applied to glaucoma.

Although many studies are still inconclusive, the potential of neuroprotective agents of glatiramer acetate, immunomodulators as well as gene and stem cell therapy as adjuvant therapy to IOP lowering agents in glaucoma should not be disregarded all together. Further research for continued understanding of these agents should persist in the hope that this will create a paradigm shift for the future of glaucoma.

## Conclusions

It has become increasingly obvious that glaucoma represents a complex multifactorial disease that produces an accelerated rate of ganglion cell atrophy related to a consortium of pathogenic mechanisms that not only most certainly involve IOP but also include defective autoregulation and ischemia, neurotrophic factor deficiency, glutamate-mediated excitotoxicity, immune-related phenomenon, weak collagenous support at the lamina cribrosa, intracellular calcium influx, and free radical damage. IOP lowering will continue to be the mainstay treatment for glaucoma. The question of alternative non-IOP-lowering therapies directed at preventing further progression has become of interest to both the desperate patient and the treating physician. Based on new, emerging research, neuroprotection has promise for preventing RGC death, independent of IOP. It is evident that pharmacological neuroprotection for glaucoma without a doubt represents an exciting development in the pursuit for a treatment modality for this debilitating disease.

## References

[1] (1994). The Advanced Glaucoma Intervention Study (AGIS): 1. Study design and methods and baseline characteristics of study patients. Control Clin Trials.

[2] (2000). The advanced glaucoma intervention study, 6: Effect of cataract on visual field and visual acuity. The AGIS Investigators. Arch Ophthalmol.

[3] (1998). Comparison of glaucomatous progression between untreated patients with normal-tension glaucoma and patients with therapeutically reduced intraocular pressures. Collaborative Normal-Tension Glaucoma Study Group. Am J Ophthalmol.

[4] Sommer A (1999). Collaborative normal-tension glaucoma study. Am J Ophthalmol.

[5] Lichter PR, Musch DC, Gillespie BW, Guire KE, Janz NK, Wren PA (2001). Interim clinical outcomes in the Collaborative Initial Glaucoma Treatment Study comparing initial treatment randomized to medications or surgery. Ophthalmology.

[6] Heijl A, Leske MC, Bengtsson B, Hyman L, Hussein M (2002). Reduction of intraocular pressure and glaucoma progression: Results from the Early Manifest Glaucoma Trial. Arch Ophthalmol.

[7] Weinreb RN, Levin LA (1999). Is neuroprotection a viable therapy for glaucoma?. Arch Ophthalmol.

[8] Weinreb RN (2006). Glaucoma neuroprotection.

[9] Kaushik S, Pandav SS, Ram J (2003). Neuroprotection in glaucoma. J Postgrad Med.

[10] Levin LA (2001). Relevance of the site of injury of glaucoma to neuroprotective strategies. Surv Ophthalmol.

[11] Gupta N, Yucel YH (2007). Should we treat the brain in glaucoma?. Can J Ophthalmol.

[12] Arundine M, Tymianski M (2003). Molecular mechanisms of calcium-dependent neurodegeneration in excitotoxicity. Cell Calcium.

[13] Sucher NJ, Lipton SA, Dreyer EB (1997). Molecular basis of glutamate toxicity in retinal ganglion cells. Vision Res.

[14] Vorwerk CK, Lipton SA, Zurakowski D, Hyman BT, Sabel BA, Dreyer EB (1996). Chronic low-dose glutamate is toxic to retinal ganglion cells. Toxicity blocked by memantine. Invest Ophthalmol Vis Sci.

[15] McKinnon SJ (1997). Glaucoma, apoptosis, and neuroprotection. Curr Opin Ophthalmol.

[16] Quigley HA, McKinnon SJ, Zack DJ, Pease ME, Kerrigan-Baumrind LA, Kerrigan DF (2000). Retrograde axonal transport of BDNF in retinal ganglion cells is blocked by acute IOP elevation in rats. Invest Ophthalmol Vis Sci.

[17] Pease ME, McKinnon SJ, Quigley HA, Kerrigan-Baumrind LA, Zack DJ (2000). Obstructed axonal transport of BDNF and its receptor TrkB in experimental glaucoma. Invest Ophthalmol Vis Sci.

[18] Raff MC (1992). Social controls on cell survival and cell death. Nature.

[19] Raff MC, Barres BA, Burne JF, Coles HS, Ishizaki Y, Jacobson MD (1993). Programmed cell death and the control of cell survival: Lessons from the nervous system. Science.

[20] Huang EJ, Reichardt LF (2003). Trk receptors: Roles in neuronal signal transduction. Annu Rev Biochem.

[21] Chau RM, Ren F, Huang WQ (1992). Programmed cell death of neonatal rat retinal ganglion cells due to turn-off expression of a novel 30-kD trophic factor and/or the lack of this factor supplied from the superior colliculus. Ann N Y Acad Sci.

[22] Nurcombe V, Bennett MR (1981). Embryonic chick retinal ganglion cells identified “*in vitro*”.Their survival is dependent on a factor from the optic tectum. Exp Brain Res.

[23] Rabacchi SA, Ensini M, Bonfanti L, Gravina A, Maffei L (1994). Nerve growth factor reduces apoptosis of axotomized retinal ganglion cells in the neonatal rat. Neuroscience.

[24] Thoenen H, Barde YA, Davies AM, Johnson JE (1987). Neurotrophic factors and neuronal death. Ciba Found Symp.

[25] Ginty DD, Segal RA (2002). Retrograde neurotrophin signaling: Trk-ing along the axon. Curr Opin Neurobiol.

[26] Quigley HA (1999). Neuronal death in glaucoma. Prog Retin Eye Res.

[27] Carpenter P, Sefton AJ, Dreher B, Lim WL (1986). Role of target tissue in regulating the development of retinal ganglion cells in the albino rat: Effects of kainate lesions in the superior colliculus. J Comp Neurol.

[28] Pearson HE, Stoffler DJ (1992). Retinal ganglion cell degeneration following loss of postsynaptic target neurons in the dorsal lateral geniculate nucleus of the adult cat. Exp Neurol.

[29] Pearson HE, Thompson TP (1993). Atrophy and degeneration of ganglion cells in central retina following loss of postsynaptic target neurons in the dorsal lateral geniculate nucleus of the adult cat. Exp Neurol.

[30] Lipton SA, Nicotera P (1998). Calcium, free radicals and excitotoxins in neuronal apoptosis. Cell Calcium.

[31] Leung CK, Lindsey JD, Crowston JG, Lijia C, Chiang S, Weinreb RN (2008). Longitudinal profile of retinal ganglion cell damage after optic nerve crush with blue-light confocal scanning laser ophthalmoscopy. Invest Ophthalmol Vis Sci.

[32] Adams JM, Cory S (1998). The Bcl-2 protein family: Arbiters of cell survival. Science.

[33] Merry DE, Korsmeyer SJ (1997). Bcl-2 gene family in the nervous system. Annu Rev Neurosci.

[34] Tatton WG, Chalmers-Redman RM, Sud A, Podos SM, Mittag TW (2001). Maintaining mitochondrial membrane impermeability. an opportunity for new therapy in glaucoma?. Surv Ophthalmol.

[35] Malik JM, Shevtsova Z, Bahr M, Kugler S (2005). Long-term in vivo inhibition of CNS neurodegeneration by Bcl-XL gene transfer. Mol Ther.

[36] Libby RT, Li Y, Savinova OV, Barter J, Smith RS, Nickells RW (2005). Susceptibility to neurodegeneration in a glaucoma is modified by Bax gene dosage. PLoS Genet.

[37] Hanninen VA, Pantcheva MB, Freeman EE, Poulin NR, Grosskreutz CL (2002). Activation of caspase 9 in a rat model of experimental glaucoma. Curr Eye Res.

[38] McKinnon SJ, Lehman DM, Kerrigan-Baumrind LA, Merges CA, Pease ME, Kerrigan DF (2002). Caspase activation and amyloid precursor protein cleavage in rat ocular hypertension. Invest Ophthalmol Vis Sci.

[39] McKinnon SJ, Lehman DM, Tahzib NG, Ransom NL, Reitsamer HA, Liston P (2002). Baculoviral IAP repeat-containing-4 protects optic nerve axons in a rat glaucoma model. Mol Ther.

[40] Levin LA WR, Di Polo A (2007). A Pocket Guide of Neuroprotection in Glaucoma.

[41] Das A, Garner DP, Del Re AM, Woodward JJ, Kumar DM, Agarwal N (2006). Calpeptin provides functional neuroprotection to rat retinal ganglion cells following Ca2+ influx. Brain Res.

[42] Tsai DC, Hsu WM, Chou CK, Chen SJ, Peng CH, Chi CW (2002). Significant variation of the elevated nitric oxide levels in aqueous humor from patients with different types of glaucoma. Ophthalmologica.

[43] Neufeld AH, Hernandez MR, Gonzalez M (1997). Nitric oxide synthase in the human glaucomatous optic nerve head. Arch Ophthalmol.

[44] Motallebipour M, Rada-Iglesias A, Jansson M, Wadelius C (2005). The promoter of inducible nitric oxide synthase implicated in glaucoma based on genetic analysis and nuclear factor binding. Mol Vis.

[45] Yuan L, Neufeld AH (2001). Activated microglia in the human glaucomatous optic nerve head. J Neurosci Res.

[46] Liu B, Neufeld AH (2000). Expression of nitric oxide synthase-2 (NOS-2) in reactive astrocytes of the human glaucomatous optic nerve head. Glia.

[47] Aslan M, Yucel I, Akar Y, Yucel G, Ciftcioglu MA, Sanlioglu S (2006). Nitrotyrosine formation and apoptosis in rat models of ocular injury. Free Radic Res.

[48] Blute TA, Lee MR, Eldred WD (2000). Direct imaging of NMDA-stimulated nitric oxide production in the retina. Vis Neurosci.

[49] Cheon EW, Park CH, Kang SS, Cho GJ, Yoo JM, Song JK (2002). Nitric oxide synthase expression in the transient ischemic rat retina: Neuroprotection of betaxolol. Neurosci Lett.

[50] Cheon EW, Park CH, Kang SS, Cho GJ, Yoo JM (2003). Change in endothelial nitric oxide synthase in the rat retina following transient ischemia. Neuroreport.

[51] Boveris A, Chance B (1973). The mitochondrial generation of hydrogen peroxide. General properties and effect of hyperbaric oxygen. Biochem J.

[52] Yildirim O, Ateş NA, Ercan B, Muşlu N, Unlü A, Tamer L (2005). Role of oxidative stress enzymes in open-angle glaucoma. Eye.

[53] Hara MR, Snyder SH (2007). Cell signaling and neuronal death. Annu Rev Pharmacol Toxicol.

[54] Wang HG, Pathan N, Ethell IM, Krajewski S, Yamaguchi Y, Shibasaki F (1999). Ca2+-induced apoptosis through calcineurin dephosphorylation of Bad. Science.

[55] Huang W, Fileta JB, Dobberfuhl A, Filippopolous T, Guo Y, Kwon G (2005). Calcineurin cleavage is triggered by elevated intraocular pressure, and calcineurin inhibition blocks retinal ganglion cell death in experimental glaucoma. Proc Natl Acad Sci U S A.

[56] Guo L, Moss SE, Alexander RA, Ali RR, Fitzke FW, Cordeiro MF (2005). Retinal ganglion cell apoptosis in glaucoma is related to intraocular pressure and IOP-induced effects on extracellular matrix. Invest Ophthalmol Vis Sci.

[57] Zhang X, Cheng M, Chintala SK (2004). Kainic acid-mediated upregulation of matrix metalloproteinase-9 promotes retinal degeneration. Invest Ophthalmol Vis Sci.

[58] Tezel G, Yang J, Wax MB (2004). Heat shock proteins, immunity and glaucoma. Brain Res Bull.

[59] Moalem G, Gdalyahu A, Shani Y, Otten U, Lazarovici P, Cohen IR (2000). Production of neurotrophins by activated T cells: Implications for neuroprotective autoimmunity. J Autoimmun.

[60] Moalem G, Leibowitz-Amit R, Yoles E, Mor F, Cohen IR, Schwartz M (1999). Autoimmune T cells protect neurons from secondary degeneration after central nervous system axotomy. Nat Med.

[61] Schori H, Kipnis J, Yoles E, WoldeMussie E, Ruiz G, Wheeler LA (2001). Vaccination for protection of retinal ganglion cells against death from glutamate cytotoxicity and ocular hypertension: Implications for glaucoma. Proc Natl Acad Sci U S A.

[62] Kipnis J, Yoles E, Porat Z, Cohen A, Mor F, Sela M (2000). T cell immunity to copolymer 1 confers neuroprotection on the damaged optic nerve: Possible therapy for optic neuropathies. Proc Natl Acad Sci U S A.

[63] Gass A, Flammer J, Linder L, Romerio SC, Gasser P, Haefeli WE (1997). Inverse correlation between endothelin-1-induced peripheral microvascular vasoconstriction and blood pressure in glaucoma patients. Graefes Arch Clin Exp Ophthalmol.

[64] Tezel G, Kass MA, Kolker AE, Becker B, Wax MB (1997). Plasma and aqueous humor endothelin levels in primary open-angle glaucoma. J Glaucoma.

[65] Wirostko B, Ehrlich R, Harris A (2009). The vascular theory in glaucoma. Glaucoma Today.

[66] Tezel G (2008). TNF-alpha signaling in glaucomatous neurodegeneration. Prog Brain Res.

[67] Barkana Y, Belkin M (2004). Neuroprotection in ophthalmology: A review. Brain Res Bull.

[68] Nouri-Mahdavi K, Hoffman D, Coleman AL, Liu G, Li G, Gaasterland D (2004). Predictive factors for glaucomatous visual field progression in the Advanced Glaucoma Intervention Study. Ophthalmology.

[69] Asrani S, Zeimer R, Wilensky J, Gieser D, Vitale S, Lindenmuth K (2000). Large diurnal fluctuations in intraocular pressure are an independent risk factor in patients with glaucoma. J Glaucoma.

[70] Chaudhary P, Ahmed F, Sharma SC (1998). MK801-a neuroprotectant in rat hypertensive eyes. Brain Res.

[71] Russo R, Cavaliere F, Berliocchi L, Nucci C, Gliozzi M, Mazzei C (2008). Modulation of pro-survival and death-associated pathways under retinal ischemia/reperfusion: Effects of NMDA receptor blockade. J Neurochem.

[72] Seif el Nasr M, Peruche B, Rossberg C, Mennel HD, Krieglstein J (1990). Neuroprotective effect of memantine demonstrated in vivo and in vitro. Eur J Pharmacol.

[73] Lipton SA (1993). Prospects for clinically tolerated NMDA antagonists: Open-channel blockers and alternative redox states of nitric oxide. Trends Neurosci.

[74] Kim TW, Kim DM, Park KH, Kim H (2002). Neuroprotective effect of memantine in a rabbit model of optic nerve ischemia. Korean J Ophthalmol.

[75] Osborne NN (2009). Recent clinical findings with memantine should not mean that the idea of neuroprotection in glaucoma is abandoned. Acta Ophthalmol.

[76] Chidlow G, Wood JP, Casson RJ (2007). Pharmacological neuroprotection for glaucoma. Drugs.

[77] Peinado-Ramon P, Salvador M, Villegas-Perez MP, Vidal-Sanz M (1996). Effects of axotomy and intraocular administration of NT-4, NT-3, and brain-derived neurotrophic factor on the survival of adult rat retinal ganglion cells. A quantitative *in vivo* study. Invest Ophthalmol Vis Sci.

[78] Mansour-Robaey S, Clarke DB, Wang YC, Bray GM, Aguayo AJ (1994). Effects of ocular injury and administration of brain-derived neurotrophic factor on survival and regrowth of axotomized retinal ganglion cells. Proc Natl Acad Sci U S A.

[79] Ji JZ, Elyaman W, Yip HK, Lee VW, Yick LW, Hugon J (2004). CNTF promotes survival of retinal ganglion cells after induction of ocular hypertension in rats: The possible involvement of STAT3 pathway. Eur J Neurosci.

[80] Fu QL, Li X, Yip HK, Shao Z, Wu W, Mi S (2009). Combined effect of brain-derived neurotrophic factor and LINGO-1 fusion protein on long-term survival of retinal ganglion cells in chronic glaucoma. Neuroscience.

[81] Nickells RW (1999). Apoptosis of retinal ganglion cells in glaucoma: An update of the molecular pathways involved in cell death. Surv Ophthalmol.

[82] Zhou X, Li F, Kong L, Tomita H, Li C, Cao W (2005). Involvement of inflammation, degradation, and apoptosis in a mouse model of glaucoma. J Biol Chem.

[83] Brookes PS, Yoon Y, Robotham JL, Anders MW, Sheu SS (2004). Calcium, ATP, and ROS: A mitochondrial love-hate triangle. Am J Physiol Cell Physiol.

[84] Osborne NN, Wood JP, Chidlow G, Bae JH, Melena J, Nash MS (1999). Ganglion cell death in glaucoma: What do we really know?. Br J Ophthalmol.

[85] Osborne NN (2008). Pathogenesis of ganglion “cell death” in glaucoma and neuroprotection: Focus on ganglion cell axonal mitochondria. Prog Brain Res.

[86] Tatton W, Chen D, Chalmers-Redman R, Wheeler L, Nixon R, Tatton N (2003). Hypothesis for a common basis for neuroprotection in glaucoma and Alzheimer’s disease: Anti-apoptosis by alpha-2-adrenergic receptor activation. Surv Ophthalmol.

[87] Chaudhary P, Ahmed F, Quebada P, Sharma SC (1999). Caspase inhibitors block the retinal ganglion cell death following optic nerve transection. Brain Res Mol Brain Res.

[88] Kermer P, Klocker N, Labes M, Bahr M (1998). Inhibition of CPP32-like proteases rescues axotomized retinal ganglion cells from secondary cell death in vivo. J Neurosci.

[89] Kermer P, Klocker N, Labes M, Thomsen S, Srinivasan A, Bahr M (1999). Activation of caspase-3 in axotomized rat retinal ganglion cells in vivo. FEBS Lett.

[90] Vidal-Sanz M, Lafuente M, Sobrado-Calvo P, Selles-Navarro I, Rodriguez E, Mayor-Torroglosa S (2000). Death and neuroprotection of retinal ganglion cells after different types of injury. Neurotox Res.

[91] Kurimoto T, Miyoshi T, Suzuki A, Yakura T, Watanabe M, Mimura O (2003). Apoptotic death of beta cells after optic nerve transection in adult cats. J Neurosci.

[92] Govindarajan B, Laird J, Sherman R, Salomon RG, Bhattacharya SK (2008). Neuroprotection in glaucoma using calpain-1 inhibitors: Regional differences in calpain-1 activity in the trabecular meshwork, optic nerve and implications for therapeutics. CNS Neurol Disord Drug Targets.

[93] Neufeld AH, Das S, Vora S, Gachie E, Kawai S, Manning PT (2002). A prodrug of a selective inhibitor of inducible nitric oxide synthase is neuroprotective in the rat model of glaucoma. J Glaucoma.

[94] Kashiwagi K, Iizuka Y, Tsukahara S (2002). Neuroprotective effects of nipradilol on purified cultured retinal ganglion cells. J Glaucoma.

[95] Nakazawa T, Tomita H, Yamaguchi K, Sato Y, Shimura M, Kuwahara S (2002). Neuroprotective effect of nipradilol on axotomized rat retinal ganglion cells. Curr Eye Res.

[96] Pang IH, Johnson EC, Jia L, Cepurna WO, Shepard AR, Hellberg MR (2005). Evaluation of inducible nitric oxide synthase in glaucomatous optic neuropathy and pressure-induced optic nerve damage. Invest Ophthalmol Vis Sci.

[97] Aydemir O, Naziroglu M, Celebi S, Yilmaz T, Kukner AS (2004). Antioxidant effects of alpha-, gamma- and succinate-tocopherols in guinea pig retina during ischemia-reperfusion injury. Pathophysiology.

[98] Dilsiz N, Sahaboglu A, Yildiz MZ, Reichenbach A (2006). Protective effects of various antioxidants during ischemia-reperfusion in the rat retina. Graefes Arch Clin Exp Ophthalmol.

[99] Janssens D, Michiels C, Delaive E, Eliaers F, Drieu K, Remacle J (1995). Protection of hypoxia-induced ATP decrease in endothelial cells by ginkgo biloba extract and bilobalide. Biochem Pharmacol.

[100] Netland PA, Chaturvedi N, Dreyer EB (1993). Calcium channel blockers in the management of low-tension and open-angle glaucoma. Am J Ophthalmol.

[101] Osborne NN, Chidlow G, Wood JP, Schmidt KG, Casson R, Melena J (2001). Expectations in the treatment of retinal diseases: Neuroprotection. Curr Eye Res.

[102] Yang H, Hirooka K, Fukuda K, Shiraga F (2009). Neuroprotective Effects of Angiotensin II Type 1 Receptor Blocker in a Rat Model of Chronic Glaucoma. Invest Ophthalmol Vis Sci.

[103] Lam TT, Fu J, Hrynewycz M, Tso MO (1995). The effect of aurintricarboxylic acid, an endonuclease inhibitor, on ischemia/reperfusion damage in rat retina. J Ocul Pharmacol Ther.

[104] Butovsky O, Koronyo-Hamaoui M, Kunis G, Ophir E, Landa G, Cohen H (2006). Glatiramer acetate fights against Alzheimer’s disease by inducing dendritic-like microglia expressing insulin-like growth factor 1. Proc Natl Acad Sci U S A.

[105] Yin Y, Cui Q, Li Y, Irwin N, Fischer D, Harvey AR (2003). Macrophage-derived factors stimulate optic nerve regeneration. J Neurosci.

[106] Simard AR, Soulet D, Gowing G, Julien JP, Rivest S (2006). Bone marrow-derived microglia play a critical role in restricting senile plaque formation in Alzheimer’s disease. Neuron.

[107] Butovsky O, Hauben E, Schwartz M (2001). Morphological aspects of spinal cord autoimmune neuroprotection: Colocalization of T cells with B7--2 (CD86) and prevention of cyst formation. FASEB J.

[108] Butovsky O, Talpalar AE, Ben-Yaakov K, Schwartz M (2005). Activation of microglia by aggregated beta-amyloid or lipopolysaccharide impairs MHC-II expression and renders them cytotoxic whereas IFN-gamma and IL-4 render them protective. Mol Cell Neurosci.

[109] Barouch R, Schwartz M (2002). Autoreactive T cells induce neurotrophin production by immune and neural cells in injured rat optic nerve: Implications for protective autoimmunity. FASEB J.

[110] Shaked I, Tchoresh D, Gersner R, Meiri G, Mordechai S, Xiao X (2005). Protective autoimmunity: Interferon-gamma enables microglia to remove glutamate without evoking inflammatory mediators. J Neurochem.

[111] Kipnis J, Cardon M, Avidan H, Lewitus GM, Mordechay S, Rolls A (2004). Dopamine, through the extracellular signal-regulated kinase pathway, downregulates CD4+CD25+ regulatory T-cell activity: Implications for neurodegeneration. J Neurosci.

[112] Kipnis J, Mizrahi T, Hauben E, Shaked I, Shevach E, Schwartz M (2002). Neuroprotective autoimmunity: Naturally occurring CD4+CD25+ regulatory T cells suppress the ability to withstand injury to the central nervous system. Proc Natl Acad Sci U S A.

[113] Ishii Y, Kwong JM, Caprioli J (2003). Retinal ganglion cell protection with geranylgeranylacetone, a heat shock protein inducer, in a rat glaucoma model. Invest Ophthalmol Vis Sci.

[114] Bull ND, Johnson TV, Martin KR (2008). Stem cells for neuroprotection in glaucoma. Prog Brain Res.

[115] Frank T, Schlachetzki JC, Göricke B, Meuer K, Rohde G, Dietz GP (2009). Both systemic and local application of granulocyte-colony stimulating factor (G-CSF) is neuroprotective after retinal ganglion cell axotomy. BMC Neurosci.

[116] Bull ND, Irvine KA, Franklin RJ, Martin KR (2009). Transplanted oligodendrocyte precursor cells reduce neurodegeneration in a model of glaucoma. Invest Ophthalmol Vis Sci.

[117] Kong GY, Van Bergen NJ, Trounce IA, Crowston JG (2009). Mitochondrial dysfunction and glaucoma. J Glaucoma.

[118] Schober MS, Chidlow G, Wood JP, Casson RJ (2008). Bioenergetic-based neuroprotection and glaucoma. Clin Experiment Ophthalmol.

[119] Wheeler DS, Jensen RA, Poss WB (2001). A randomized, blinded comparison of chloral hydrate and midazolam sedation in children undergoing echocardiography. Clin Pediatr (Phila).

[120] Kalapesi FB, Coroneo MT, Hill MA (2005). Human ganglion cells express the alpha-2 adrenergic receptor: Relevance to neuroprotection. Br J Ophthalmol.

[121] Gao H, Qiao X, Cantor LB, WuDunn D (2002). Up-regulation of brain-derived neurotrophic factor expression by brimonidine in rat retinal ganglion cells. Arch Ophthalmol.

[122] Donello JE, Padillo EU, Webster ML, Wheeler LA, Gil DW (2001). Alpha(2)-Adrenoceptor agonists inhibit vitreal glutamate and aspartate accumulation and preserve retinal function after transient ischemia. J Pharmacol Exp Ther.

[123] Wheeler LA, Tatton NA, Elstner M, al e (2001). Alpha-2 adrenergic receptor activation by brimonidine reduces neuronal apoptosis through Akt (Protein Kinase B) dependent new synthesis of BCL-2. Invest Ophthalmol Vis Sci.

[124] Ballif BA, Blenis J (2001). Molecular mechanisms mediating mammalian mitogen-activated protein kinase (MAPK) kinase (MEK)-MAPK cell survival signals. Cell Growth Differ.

[125] Xia Z, Dickens M, Raingeaud J, Davis RJ, Greenberg ME (1995). Opposing effects of ERK and JNK-p38 MAP kinases on apoptosis. Science.

[126] Dong CJ, Guo Y, Agey P, Wheeler L, Hare WA (2008). Alpha2 adrenergic modulation of NMDA receptor function as a major mechanism of RGC protection in experimental glaucoma and retinal excitotoxicity. Invest Ophthalmol Vis Sci.

[127] Krupin T, Liebmann JM, Greenfield DS, Rosenberg LF, Ritch R, Yang JW (2005). The Low-pressure Glaucoma Treatment Study (LoGTS) study design and baseline characteristics of enrolled patients. Ophthalmology.

[128] Osborne NN, Wood JP, Chidlow G (2005). Invited review: Neuroprotective properties of certain beta-adrenoceptor antagonists used for the treatment of glaucoma. J Ocul Pharmacol Ther.

[129] Baptiste DC, Hartwick AT, Jollimore CA, Baldridge WH, Chauhan BC, Tremblay F (2002). Comparison of the neuroprotective effects of adrenoceptor drugs in retinal cell culture and intact retina. Invest Ophthalmol Vis Sci.

[130] Gross RL, Hensley SH, Gao F, Wu SM (1999). Retinal ganglion cell dysfunction induced by hypoxia and glutamate: Potential neuroprotective effects of beta-blockers. Surv Ophthalmol.

[131] Hirooka K, Kelly ME, Baldridge WH, Barnes S (2000). Suppressive actions of betaxolol on ionic currents in retinal ganglion cells may explain its neuroprotective effects. Exp Eye Res.

[132] Melena J, Stanton D, Osborne NN (2001). Comparative effects of antiglaucoma drugs on voltage-dependent calcium channels. Graefes Arch Clin Exp Ophthalmol.

[133] Zhang J, Wu SM, Gross RL (2003). Effects of beta-adrenergic blockers on glutamate-induced calcium signals in adult mouse retinal ganglion cells. Brain Res.

[134] Chidlow G, Melena J, Osborne NN (2000). Betaxolol, a beta(1)-adrenoceptor antagonist, reduces Na(+) influx into cortical synaptosomes by direct interaction with Na(+) channels: Comparison with other beta-adrenoceptor antagonists. Br J Pharmacol.

[135] Melena J, Wood JP, Osborne NN (1999). Betaxolol, a beta1-adrenoceptor antagonist, has an affinity for L-type Ca2+ channels. Eur J Pharmacol.

[136] Tan AY, LeVatte TL, Archibald ML, Tremblay F, Kelly ME, Chauhan BC (2002). Timolol concentrations in rat ocular tissues and plasma after topical and intraperitoneal dosing. J Glaucoma.

[137] Osborne NN, DeSantis L, Bae JH, Ugarte M, Wood JP, Nash MS (1999). Topically applied betaxolol attenuates NMDA-induced toxicity to ganglion cells and the effects of ischaemia to the retina. Exp Eye Res.

[138] DeSantis L, Dahlin D, Barnes G, Drance S (1995). A role for the calcium channel blocking-like action of betaxolol for providing therapeutic benefit to glaucoma patients. Update to glaucoma, ocular blood flow, and drug treatment.

[139] Goto W, Ota T, Morikawa N, Otori Y, Hara H, Kawazu K (2002). Protective effects of timolol against the neuronal damage induced by glutamate and ischemia in the rat retina. Brain Res.

[140] Messmer C, Flammer J, Stumpfig D (1991). Influence of betaxolol and timolol on the visual fields of patients with glaucoma. Am J Ophthalmol.

[141] Collignon-Brach J (1992). Long-term effect of ophthalmic beta-adrenoceptor antagonists on intraocular pressure and retinal sensitivity in primary open-angle glaucoma. Curr Eye Res.

[142] Collignon-Brach J (1994). Longterm effect of topical beta-blockers on intraocular pressure and visual field sensitivity in ocular hypertension and chronic open-angle glaucoma. Surv Ophthalmol.

[143] Kaiser HJ, Flammer J, Stumpfig D, Hendrickson P (1994). Longterm visual field follow-up of glaucoma patients treated with beta-blockers. Surv Ophthalmol.

[144] Drance SM (1998). A comparison of the effects of betaxolol, timolol, and pilocarpine on visual function in patients with open-angle glaucoma. J Glaucoma.

[145] Vainio-Jylha E, Vuori ML (1999). The favorable effect of topical betaxolol and timolol on glaucomatous visual fields: A 2-year follow-up study. Graefes Arch Clin Exp Ophthalmol.

[146] Araie M, Azuma I, Kitazawa Y (2003). Influence of topical betaxolol and timolol on visual field in Japanese open-angle glaucoma patients. Jpn J Ophthalmol.

[147] Rainer G, Dorner GT, Garhofer G, Vass C, Pfleger T, Schmetterer L (2003). Changing antiglaucoma therapy from timolol to betaxolol: Effect on ocular blood flow. Ophthalmologica.

[148] Miki H, Miki K (2004). The effects on the intraocular pressure and visual field resulting from a switch in the treatment from timolol to betaxolol. J Ocul Pharmacol Ther.

[149] Kudo H, Nakazawa T, Shimura M, Takahashi H, Fuse N, Kashiwagi K (2006). Neuroprotective effect of latanoprost on rat retinal ganglion cells. Graefes Arch Clin Exp Ophthalmol.

[150] Drago F, Valzelli S, Emmi I, Marino A, Scalia CC, Marino V (2001). Latanoprost exerts neuroprotective activity *in vitro* and *in vivo*. Exp Eye Res.

[151] Melamed S (2002). Neuroprotective properties of a synthetic docosanoid, unoprostone isopropyl: Clinical benefits in the treatment of glaucoma. Drugs Exp Clin Res.

[152] Hare WA, WoldeMussie E, Weinreb RN, Ton H, Ruiz G, Wijono M (2004). Efficacy and safety of memantine treatment for reduction of changes associated with experimental glaucoma in monkey, II: Structural measures. Invest Ophthalmol Vis Sci.

[153] Fuchsjäger-Mayrl G, Wally B, Rainer G, Buehl W, Aggermann T, Kolodjaschna J (2005). Effect of dorzolamide and timolol on ocular blood flow in patients with primary open angle glaucoma and ocular hypertension. Br J Ophthalmol.

[154] Seki M, Tanaka T, Matsuda H, Togano T, Hashimoto K, Ueda J (2005). Topically administered timolol and dorzolamide reduce intraocular pressure and protect retinal ganglion cells in a rat experimental glaucoma model. Br J Ophthalmol.

[155] Sarup V, McEwan GC, Thompson C, Patil KA, Sharma SC (2005). Dorzolamide and timolol saves retinal ganglion cells in glaucomatous adult rats. J Ocul Pharmacol Ther.

[156] Tsai JC, Song BJ, Wu L, Forbes M (2007). A candidate neuroprotective agent in the treatment of glaucoma. J Glaucoma.

[157] Ritch R (2007). Natural compounds: Evidence for a protective role in eye disease. Can J Ophthalmol.

[158] Castro-Sanchez AM, Moreno-Lorenzo C, Mataran-Penarrocha GA, Feriche-Fernandez-Castanys B, Granados-Gamez G, Quesada-Rubio JM Connective Tissue Reflex Massage for Type 2 Diabetic Patients with Peripheral Arterial Disease: Randomized Controlled Trial. Evid Based Complement Alternat Med 2009.

[159] Kusari J, Zhou S, Padillo E, Clarke KG, Gil DW (2007). Effect of memantine on neuroretinal function and retinal vascular changes of streptozotocin-induced diabetic rats. Invest Ophthalmol Vis Sci.

[160] Wilhelm B, Ludtke H, Wilhelm H (2006). Efficacy and tolerability of 0.2% brimonidine tartrate for the treatment of acute non-arteritic anterior ischemic optic neuropathy (NAION): A 3-month, double-masked, randomised, placebo-controlled trial. Graefes Arch Clin Exp Ophthalmol.

[161] Weinreb RN, Gupta N, Girkin C, Goldberg I (2006). Workshop breakout group.

